# Supplementary material for: Adjusting the 15-method to Danish general practice: a participatory design approach
Source: Scand J Prim Health Care. 2026 Jan 2;44(1):2606046. doi: 10.1080/02813432.2025.2606046 (PMC12777768; doi:10.1080/02813432.2025.2606046)
Supplement: Supplementary File 1_Description of workshops.docx [file IPRI_A_2606046_SM4270.docx]

# Workshop 1

Four sessions of twenty minutes.

Session one was a body-storming exercise on how to address alcohol habits in a non-judgmental way in a primary care setting. Body-storming is a method of enacting, brainstorming, and prototyping in context on a specific issue or topic (1, 2) and allows for making sense of a problem or situation and simulating solutions to these in real-time. By enacting the scenario of inquiring about alcohol habits, the participants could try out different ways of framing a question or sentence and experience the scenario firsthand. The bodily knowledge gained by a design team has been argued as an essential basis for generating ideas and making proposals for change (3) and the participants were instructed to take turns being the one asking the questions. Two participants at a time enacted a scenario with the opportunity to pause or rewind the conversation, while the remaining group members took notes. Between the “conversations” the group reflected on their notes and wrote phrases and reflection on a white board. The room was furnished as a general practice consultation and the session was facilitated by the behavioral psychologist.

The second session took place in a room arranged as a design studio with a stand-up working area furnished with tables and whiteboards. The graphic designer facilitated the session and focused on the 15-method’s material. The graphic designer used a Who-What-Where-How matrix (who is the material for, what should the material consist of, where should the material be applied/be accessible, and how should it be used). In groups of two, the participants were instructed to spend approximately five minutes in each quadrant writing down notes on post-its. The session concluded with a brief presentation by the participants on their notes and insights. The technique is inspired by the “4 C’s” (components, characteristics, challenges, and characters) by Gray, Brown, and Macanufo (4).

The third session was a brain-writing exercise (5) on how to best make HCPs and patients prioritize the time and resources needed to use the 15-method in a general practice setting. Brain-writing is a combined reflective writing and brainstorming exercise in which each participant starts with a piece of paper, writes down an idea, and passes the paper to the next person, who then either tries to elaborate on this idea, or writes a new idea. The participants collectively try to answer or come up with ideas to a specific question, in this case the aspect of interdisciplinary work, opportunities to implement the 15-method, and how to make the best use of the method’s homework assignments. In this scenario, notes were passed around the table for approximately 10 minutes before a new question was put forth for the participants to focus on. The room was furnished as a lounge area with couches and coffee tables, and the session was facilitated by PNS.

The fourth session was a concept development speed round to envision the optimal future solution for treating alcohol problems in a primary care setting. The applied technique was based on “3-12-3 brainstorm” by Macanufo (4) and Future Workshops (6). The participants started off with three minutes of free association and writing down notes and then progress to develop the concept in pairs from their individual notes. Finally, the pairs made a brief three-minute presentation on their thinking to the other groups, highlighting the note or thought that influenced their thinking the most. If participants felt stuck, the technique “5 Why’s” (7) was encouraged, in which the participants repeatedly ask the question “why” to move the process along. The room was furnished as a boardroom meeting with tables and white-boards and was facilitated by the project manager from the Health Innovation Centre.

# Workshop 2

Three sessions of thirty minutes followed by plenary discussion.

The three patient sessions
The first session focused on how the material could help tie consultations together in a general practice setting. The format was a group discussion conducted in a scenario-based setting of a general practice office to simulate a consultation. The scenario-based setting was chosen to help stimulate visions for future use of the material and to enable common reflections between the participants and development team (8). PNS facilitated the discussion and focused on when or whether the patients would prefer to receive hand-out material and how much material was considered suitable for different consultations. The prototype material form workshop 1 was laid out on a table, and the patients were encouraged to interact with the material and provide specific feedback on the material and adjustments from workshop 1.

The patients’ second session was a discussion with the graphic designer who, based on input from workshop 1, had prepared design and layout updates between the workshops. The setting was a lounge area with a coffee table on which prototypes and mood-boards were presented for the participant to interact with. Prototyping is a core technique in PD (3) and we used mood-boards as they can help facilitate and direct a design process (9). The focus of the session was partly on the visual aspects of the material, and partly on the framing and wording within the patient material to ensure non-stigmatizing language and high reader friendliness.

The patients’ final session was a brain-writing exercise in an adjourning room. The patients received written questions on separate sheets of paper and were asked to use them as a starting point for idea generation and circulate the papers to the next person to build on each other’s answers and ideas. Questions revolved around how to make the aspect of “homework” more attractive, how, where, and when questionnaires such as the AUDIT questionnaire could be utilized, and how support from personal networks might be considered best integrated in the *15-method*.

The three healthcare professional sessions
The behavioral psychologist facilitated the first session which was a stand-up brainstorm using prototypes, white-board notes, and drawings. The goal was to create an overview of the 15-method in a quick guide format that could be used as a referencing tool to the HCP manual. The HCPs worked in two groups on layouts, how and when to include AUDIT screening, and how to present next available options for the HCP at each step in the quick guide. The HCPs could use the original Danish 15-method material and prototypes of the adjusted version to create mock-ups which were then drawn on white-boards for elaboration.

The second HCP session was a body-storming exercise to enact the workflow of their newly designed mock-up quick guide. A group first presented their mock-up to the other groups and proceeded to enact a series of short consultations in which the quick guide was tested. The project manager from the Health Innovation Centre facilitated the session and noted ideas, discussion points, and quotes on a whiteboard during the session. The session concluded with a discussion on possible adjustments and a joined mock-up summarizing the groups’ work was created on the white board.

The HCPs’ final session was a brain-writing exercise on the HCP material and the 15-method structure. The format was identical to the patients’ brain-writing exercise but the questions differed and revolved around how to make the 15-method a priority in everyday work, where to best store and retrieve material and information in the practice, how to best support patients in using the material, and how the method might help nudge HCP to inquire more frequently about alcohol habits.

1. Oulasvirta A, Kurvinen E, Kankainen T. Understanding contexts by being there: case studies in bodystorming. Personal and Ubiquitous Computing. 2003;7(2):125-34.

2. Schleicher D, Jones P, Kachur O. Bodystorming as embodied designing2010; 17(6):[47-51 pp.]. Available from: <https://go.exlibris.link/xKvwywrN>.

3. Simonsen J, Robertson T. Routledge international handbook of participatory design: Routledge New York; 2013.

4. Gray D, Brown S, Macanufo J. Gamestorming: A playbook for innovators, rulebreakers, and changemakers: " O'Reilly Media, Inc."; 2010.

5. VanGundy AB. Brain writing for new product ideas: an alternative to brainstorming. The Journal of consumer marketing. 1984;1(2):67-74.

6. Jungk R, Müllert N. Future workshops: How to create desirable futures: Inst. for Social Inventions; 1987.

7. Serrat O. The Five Whys Technique. Knowledge Solutions2017. p. 307-10.

8. Kyng M. Making representations work. Communications of the ACM. 1995;38(9):46-55.

9. Lucero A. Framing, aligning, paradoxing, abstracting, and directing: how design mood boards work. Proceedings of the Designing Interactive Systems Conference; Newcastle Upon Tyne, United Kingdom: Association for Computing Machinery; 2012. p. 438–47.
