# Supplementary material for: Adjusting the 15-method to Danish general practice: a participatory design approach
Source: Scand J Prim Health Care. 2026 Jan 2;44(1):2606046. doi: 10.1080/02813432.2025.2606046 (PMC12777768; doi:10.1080/02813432.2025.2606046)
Supplement: revised_manuscript_clean_version.docx [file IPRI_A_2606046_SM4269.docx]

# Adjusting the 15-method to Danish general practice: a participatory design approach

Peter Schøler, MD, PhD. 1, 2

Jens Søndergaard, Professor, MD, PhD, GP, Clin. Pharm. 2

Sanne Rasmussen, Associate Professor, MD, PhD. 2

Anette Søgaard Nielsen, Professor, PhD. 1, 3

1. Unit for Clinical Alcohol Research, Research Unit of Psychiatry, University of Southern Denmark, Odense, Denmark

2. Research Unit of General Practice, Department of Public Health, University of Southern Denmark, Odense, Denmark

3. BRIDGE, Brain Research - Inter Disciplinary Guided Excellence, University of Southern Denmark, Odense, Denmark

## Corresponding author

Peter Næsborg Schøler. Phone number: +45 21356266. Email: pscholer@health.sdu.dk
Address: J.B. Winsløwsvej 18, ent. 220B, first floor, DK-5000 Odense C, Denmark.

ORCiD: 0000-0002-1959-3251
Word count excluding abstract: 3252

# Abstract

## Background

The 15-method is a primary healthcare tool for opportunistic screening and brief intervention for alcohol-related problems. A Danish feasibility test of the 15-method suggested that adjustments might enhance its fit to Danish general practice. This study reports on user-involving workshops aimed at refining a Danish version of the 15-method.

## Methods

Using a participatory design approach, we conducted iterative cycles of planning, user workshops, evaluating, and revising design. Workshops engaged five general practitioners, three nurses, four patients, four researchers, a project manager, a graphics designer, and a behavioral design specialist. Through prototyping and scenario enacting, participants co-developed solutions to improve the 15-method’s structure and usability. Prototypes were field tested and evaluated.

## Results

The 15-method*’*s treatment step was shortened from four consultations to three by re-structuring of the patient material and increasing flexibility in transitioning from screening to treatment. A quick guide was developed as a shared reference for patients and healthcare professionals, alongside visual aids such as flyers and posters. The healthcare manual was re-designed for flexibility and updated to include varied screening approaches. Field testing confirmed that these adjustments enhanced usability and interdisciplinary collaboration.

## Conclusion

The participatory design process effectively refined the 15-method for Danish general practice. Future studies will assess the effectiveness of the adapted version on a larger scale.

Keywords: Alcohol Use Disorder, Primary Healthcare, Screening and Brief Intervention, Participatory Research, Physician-Patient Relations

# Introduction

Harmful alcohol causes an estimated three million deaths annually worldwide, surpassing the mortality burden of tuberculosis, HIV, diabetes, digestive diseases, and hypertension (1). Most harm is caused by hazardous and harmful use rather than severe dependence (2). Thus, early interventions for harmful and hazardous use hold potential for substantial population-level impact (3). However, the implementation of alcohol interventions in primary healthcare remains challenging (4-6). Healthcare professionals (HCPs) face barriers including lack of resources, insufficient training and support, high workload, and concerns of patient stereotyping (7, 8). Meanwhile, patient-related barriers for treatment seeking include fear of stigmatization and uncertainty about treatment options (7, 9). To address these challenges, Andréasson and colleagues developed the 15-method, a novel method for addressing and treating alcohol-related problems in primary care (10). In Swedish primary care, the 15-method was found as effective in lowering patient alcohol consumption as specialist treatment in patients with mild to moderate alcohol dependence (11). We are currently evaluating the 15-method in Danish primary care following The Medical Research Council framework for developing and evaluating complex interventions (12). Our initial feasibility study demonstrated that the method is meaningful and acceptable to both HCPs (general practitioners and nurses) and patients, though contextual adaptations were needed for Danish primary care (13). Subsequent interviews identified key barriers, facilitators, and specific method components requiring attention to optimize contextual fit (14). These findings informed areas for user workshops aimed at refining the 15-method.

The present study reports on this workshop-based adaptation process. Specifically, we 15-methodaimed to develop and finalize a Danish adapted version of the 15-method for primary care.

# Methods

## The 15-method

The method’s name refers to two aspects: it targets patients with an Alcohol Use Disorder Identification Test (AUDIT)(15) score >15, and consultations are 15 minutes. The method integrates opportunistic screening, stepped-care (16), and Motivational Interviewing (17) techniques in three steps, providing healthcare professionals with structured assessment and treatment tools for alcohol-related problems.

Step one: opportunistic screening and brief advice. During routine appointments, HCPs assess alcohol habits in relation to presenting symptoms, contact reasons, and laboratory findings. The HCP offers brief advice and information on alcohol related to the patient’s situation. The patient may fill in the AUDIT during the consultation or at home.

Step two: health-check. The HCP conducts an overall health-assessment in relation to the patient’s alcohol habits based on AUDIT score, laboratory tests, and other symptom-relevant data. Additional assessment tools include the one-week Timeline Follow-Back (18), the Short Alcohol Dependence Data Questionnaire (19), and the International Classification of Diseases 10^th^ revision (ICD-10) criteria for alcohol dependence (20), and other substance use screening when indicated (benzodiazepines, opioids). Through motivational interviewing, the HCP explores patient readiness for change and presents treatment offers, including progression to step three or specialist referral.

Step three: guided self-change treatment (21). Through homework assignments and up to three scheduled consultations, the patient and HCP work on themes such as identification of risk-situations, alternatives to drinking, goal setting, and action plans. Assignments are based on elements from Cognitive Behavioral Therapy and consultation are facilitated using Motivational Interviewing (17). Treatment may also include pharmacological aids (disulfiram, acamprosate, nalmefene, and naltrexone) per national guidelines.

## Methodology and framework

This study employed a participatory design methodology (22). Following preliminary interviews identifying adaptation requirements (14), the present study applied the “make” and “enact” phases of participatory design (23) through user workshops. We conducted iterative co-design workshops with healthcare professionals, patients, and researchers, following cycles of planning, conducting, reflecting, evaluating, and revising design (22) (Figure 1). Participatory design helps create a “third space” for mutual learning and exploration with respect for different types of knowledge (22, 24). This approach generates novel solutions via modified “rules”, roles, scenarios and tangible artifacts, in a co-realization process (22). We wanted to facilitate a meeting of languages and practices (25), in this case between HCPs, patients, and researchers, with an emphasis on power-sharing between participants (26, 27).Setting

### Danish primary care

Denmark has approximately 3500 general practitioners (GPs) in 1650 practices(28, 29). Each GP has 1500-2000 listed patients and 99% of Danish residents are listed with a GP (30, 31). The GP is self-employed and reimbursed through the tax-funded healthcare system. Consultations are free of charge for the patient, and general practice serves as a point of first-contact, as first-line provider, and as gatekeeper to the secondary healthcare sector (32).

### Workshop setting

We conducted workshops at the Health Innovation Centre of Southern Denmark who specializes in user involvement processes, service design, technological developments in healthcare, and public-private partnerships.

## The research team and development team

PNS is a medical doctor and postdoc researcher. ASN is professor in clinical alcohol research and JS is professor, general practitioner, and clinical pharmacologist.SR is associate professor and general practitioner trainee. The development team included an independent graphic designer, a behavioral psychologist with expertise in behavioral design and nudging, and PNS. The research team (ASN, JS, SR) oversaw the process and provided feedback but did not participate in the workshops.

## Participant characteristics and recruitment

All healthcare professionals and patient representatives had participated in the interview study (14) informing the present study. We sought insights from HCPs (GPs and nurses with and without experience of using the 15-method, as we wanted input on both specific details to the method’s material, and inputs on general practice in general, including what an optimal alcohol intervention might look like. HCPs were recruited through purposive sampling (33) targeting urban/rural and solo/partnership practice variation. Patient recruitment sought diverse alcohol-related problem experience and general practice familiarity, regardless of 15-method exposure, as the patient perspective revolved around initial contact, communication around alcohol, and ideal treatment. Patients were recruited via snowball sampling (33) through a user-panel at Alcohol & Society (34), an interest organization working toward healthier alcohol habits in Denmark, and through research network affiliations.

Table 1 features participant characteristics and workshop attendance. The participants comprised five GPs, three nurses, and four patients. Three GPs and one nurse had experience working with the 15-method*.* Patients’ self-reported alcohol-related problems varied from none to severe and included both current and past problems.

> Insert Table 1 about here <

## Data collection and data storage

### Data collection

Data included observation notes, reflections, white-board notes, photographs, post-its, writing exercises, drawings, and audio recordings in selected sessions. The development team collected data during and after the workshops.

### Data storage

Personable data were stored on secure serves at Odense Patient data Explorative Network (OPEN) (35), Region of Southern Denmark, in compliance with the European General Data Protection Regulations.

## Analysis

Analysis in participatory design is an intertwined and ongoing process that takes place both between and during sessions and activities. The participants are actively engaged in exploring, designing, re-iterating, and creating new understandings of the situation and each new insight or activity creates a new situation or understanding which is then in turn analyzed and discussed (36, 37).

The development team shared their reflections, notes, and collected data throughout the process and discussed the main findings in relation to the study’s overall aim in separate team meetings (detailed in Procedures and Figure 1). The development team also discussed possible reiteration, key points, or activities needed to move the process forward (22, 38) and planned upcoming activities accordingly, e.g. activities with stronger focus on concrete design details to clarify ideas raised in the workshops. The graphic designer and behavioral psychologist were free to work on ideas, prototypes, and mock-ups between sessions and communicated with the development team via e-mail and phone between team meetings.

## Procedure

The procedure comprised five development team meetings, two user-workshops, a field test of the adapted material, a feedback session with HCPs from the field test, an evaluation with the overall research team (ASN, JS, SR), and a production phase (Figure 1). The development team facilitated both user workshops with assistance from a project manager at the Health Innovation Centre. We used the participatory design procedure *Plan, Act, Observe, Reflect* presented by Clemensen et al. 2017 (39). The development team planned the workshops with different tools and techniques such as exploratory games (40), varying scenarios, and reflective exercises (22, 41).

We built on findings from our preceding interview study ,(14), which identified two key focus areas: *Communication and material*, and *Integration to workflows.* Briefly described, the former focused on condensing and lightening the HCP and patient material, restructuring their layout, and strengthening support for HCPs in addressing alcohol habits, e.g. by including phrases or example sentences. The latter focused on embedding alcohol screening into existing procedures, digitalizing material, , developing reminders, strengthening interdisciplinary work, and increasing the flexibility in the treatment modules. Additional suggestions included creating a visual overview of the method, visual aids to facilitate consultations on alcohol habits, and icebreakers for e.g. waiting room areas.

### User workshop 1

The first workshop had a duration of two hours and started with a 15-minute introduction to the format of the workshop and on the overall aim of the process. The participants were divided into three groups mixing HCP and patients. The three groups rotated through four different scenarios in a “dollhouse” format (see Figure 2) in four twenty-minute sessions with different tasks and exercises. The workshop concluded with a twenty-minute plenary session for discussion and reflections.

### User workshop 2

The second two-hour workshop began with plenary reflection on the previous workshop. Participants separated into healthcare professional and patient groups for user-specific adaptations through three parallel thirty-minute activities, concluding with plenary discussion.

Workshop activities are detailed in Supplementary File 1.

### Field test and feedback

The development team adjusted the material based on findings from the user workshops and discussed the updated material with the research team. Four healthcare professionals from the workshops with prior 15-method experience conducted a three-week field test in routine practice to provide real-use feedback (42). The HCPs provided feedback on layout, quality, user friendliness, structure, and usability through video discussions. The development team then readied the final version for production and print.

# Results

## The workshops

### The patient material

Home-work assignments, alcohol calendar, and lifestyle registrations (smoking, diet, exercise, and alcohol) were compiled into a single piece patient logbook. Participants found it helpful that the logbook served as the sole reference point throughout the 15-method. It contained motivational quotes, space for lifestyle registration, reflection pages, consultation reminders, and homework assignments. Patients preferred the name “logbook” over “diary”, which felt too personal to share with one’s doctor or nurse. Homework assignments and intermediate daily-log pages for lifestyle registration were spaced to match the suggested treatment timeline.

Three different flyers and three posters were created, each with a different approach to inform on alcohol, national guidelines, alcohol-related harm, and were to get more information and help. The material emphasized several points from the patient and HCP brain-writing sessions. First, that treatment for alcohol problems is possible in general practice. Second, the material addressed taboo and stigma directly, and third, that treatment options are flexible, and the treatment goal is individual.

The patients also considered QR codes, apps, podcasts, and social media to be valuable sources of information when looking for treatment options or information. None of these options were explored more in the present study.

### The healthcare professional material

The HCP session on workflow and method structure resulted in a two-sided quick guide with dual functions. One side presented an overview of the method’s steps and options, cross referenced with the HCP and patient materials. The guide was color coded to the HCP manual for easy reference, while quotes from the body-storming sessions and reminders for scheduling appointments were also included. The reverse side contained an infographic on physical and mental benefits of reducing alcohol consumption, providing a visual reference to facilitate discussions on alcohol habits and treatment goals (Figure 3).

The HCP manual was revised to focus more on exploring patient motivation and on offering different approaches to information-giving. This adjustment reflected a difference in perspectives: HCPs prioritized communicating risks and consequences of alcohol-related harm, assuming patients would make a rational decision. Meanwhile, the patients emphasized envisioning a healthy, meaningful life with deep relationships over distant health threats. The manual was therefore adapted to encompass both perspectives, encouraging HCPs to focus on what patients find most motivating for change.

### Structure, workflow, and additional materials

HCP and patient brain-writing sessions favored a flexible structure delivered in small “bites” with follow-up opportunities, rather than a “standard” package of questionnaires and home-work assignments. Patient could, for instance, move directly from screening (step one to a relevant home-work assignment in treatment (step three), a format illustrated in the quick guide.

The treatment step was reduced from four consultations to three, as exploration of motivation and goal setting were combined in the logbook. Both patients and HCPs valued this reduction, provided the concluding follow-up session was preserved as a review of the latest assignment, such as an action plan.

To further support flexibility, several tools from the original 15-method were moved to a “tips, tricks, and additional material” section in the HCP manual, with online access to the questionnaires. This included the Short Alcohol Dependency Data questionnaire (43), a six-item questionnaire based on the ICD-10 diagnostic criteria for alcohol dependence syndrome, the Timeline Follow Back (18), and a questionnaire on the use of tobacco, opioids, and illicit drugs. The section also offered checklists, suggested phrases for patient filing systems, and practical advice on sustaining a focus on alcohol in routine practice.

## Field test

The field test showed that icebreakers such as posters were useful and could be expanded to include info-screens or other visual prompts in waiting areas. The quick guide also functioned well, facilitating transitions between staff groups by providing a visual overview of patient progress and possible next steps.

Although none of the HCPs used the online version, they integrated the suggested phrases and checklists into their patient filing system, for example in yearly controls. The HCPs also found the quotes included in the quick guide and checklists helpful.

Examples of icebreakers are published elsewhere (44), and all materials are available in Danish at www.sdu.dk/15-metoden.

# Discussion

In this participatory design study, we finalized the Danish adapted version of the 15-method for treating alcohol-related problems in general practice.

The core elements of the 15-method, with its three steps and theoretical underpinnings in Motivational Interviewing and cognitive behavioral therapy, remain unchanged. Slight but important alterations were made to the overall structure of the method to increase its fit to Danish general practice, and the material for HCPs and patients was condensed and re-designed for the updated structure. The increased flexibility requested for the Danish 15-method likely reflects differences between treatment-seeking patient populations at Riddargatan 1, Stockholm–where the method originated(10)–and non-treatment-seeking general practice patients. For opportunistic screening contexts, the structured dialogue framework served as a common reference point and the flexible use of material proved to be an important aspect for facilitating alcohol discussions and exploring motivation for change across multiple visits.

From a public health perspective, brief alcohol interventions in primary care offer substantial population-level impact by targeting hazardous and harmful use in a patient population who typically can reduce consumption with appropriate support (45). Reduction to low-risk consumption levels translates to meaningful and long-term health improvements such as decreased mortality risk (46), improved mental health and quality of life, and reduced physiological markers including systolic blood pressure and liver enzymes (47-49). General practice holds a unique position for addressing alcohol-related harm and can provide contact with individuals unlikely to seek specialized treatment. This is critical given that mild-to-moderate alcohol problems constitute most cases but are the least likely to receive treatment (50, 51). The adapted 15-method may address part of this treatment gap through structured opportunistic screening integrated into routine consultations. Alternative low-intensity approaches have also been proposed, such as biomarker-supported screening like B-phosphatidylethanol (PEth) in relevant patient groups. While these may serve as a complementary avenue in primary care, their current use is limited and subject for continued discussion (51-53).

A large-scale effectiveness evaluation of the 15-method is currently underway (54) and contextual adaptation prior to such testing is essential from an implementation science perspective. Pre-evaluation adaptations may help increase intervention fit when transferring interventions from one context to another and increase chances of implementation success (55, 56). Without adequate adaptation prior to evaluation, effectiveness trials may produce misleading results. An intervention may appear ineffective, when the real problem is poor implementation such as incompatible workflows or sub-optimal resource allocation (57, 58), leaving causal mechanisms unclear (55). Adaptations can ensure at least two practical necessities: feasibility – can HCPs actually use the intervention with the available time and resources? And acceptability – will users (HCPs and patients) engage with it in a real-world setting? (59). The present study’s user workshops systematically addressed barriers specific to Danish general practice, optimizing the 15-method’s fit prior to large-scale evaluation. Our most recent implementation-focused evaluation (44) further shows that the adapted method addresses several barriers in real-world use. This provides a strong foundation for future implementation efforts and exemplifies how deliberate user-involvement in the adaptation process can translate high-level implementation frameworks into concrete solutions for practice. Finally, the AUDIT questionnaire remains central to the 15-method. Digitalization into patient filing systems, though identified as important, is being addressed beyond the scope of the present study.

## Methodological considerations

The participatory research methodology enabled mutual learning between users and developers, helping to increase the applicability and acceptability of the 15-method (60). We planned the workshops in a participatory design format (22), making an effort to empower participants and avoid tokenism (61), with the goal of genuinely learning from their inputs (62). The process had a defined scope and timeframe and focused on adjusting an existing method and testing new prototypes (63), while remaining faithful to the 15-method*’*s core elements as a stepped-care model (16) based on Motivational Interviewing (17) and guided self-change (21). Since the aim was to refine rather than redesign the 15-method, the scope, and to some extent the workshop agendas were set by the research and development teams. These constraints were deliberate, as refinements were needed within a limited timeframe prior to large-scale evaluation (12). Practical constraints also shaped the process as HCPs had limited time to attend workshops. The limited timeframe and constraints hold an important methodological consideration with regards to the level of power-sharing, as the participants were free to work only within the constraints of the process (27). To address this and make sure everyone had a say, we emphasized transparency by clearly stating the framework and overall process design at the outset. We also encouraged participants to raise issues outside the agenda to avoid silencing any issues or participants (64).

Participatory design often include three integrated phases, named “telling”, “making” and “enacting” (22). In the present study, we focused on making and enacting, while drawing from findings from our previous studies on feasibility and adaptation requirement (13, 14). We did not conduct a single study encompassing all three phases (tell, make, enact), out of logistical considerations, as we had participants from different parts of the country, and out of time-constraints, as discussed above. A major limitation is that no female patients participated in the workshops. This limits the generalizability of the findings and increases the risk of a gender bias in the material.

## Conclusion and future directions

The participatory design approach facilitated collaboration among healthcare professionals, patients, researchers, and developers to adapt the 15-method for Danish general practice. A large-scale effectiveness evaluation is currently underway, and future research should examine how participatory research approaches can inform scaling strategies, should effectiveness be demonstrated, and how these insights can guide sustainable implementation in primary care.

Acknowledgments
We thank Trygfonden for unconditionally funding this project and Odense Patient data Explorative Network (OPEN) for data storage and handling. We further thank the Health Innovation Centre of Southern Denmark for hosting and facilitating the workshops with special thanks to project manager Morten Givskud. Also, thanks to Henrik Nielsen (www.the-pony.com) for graphic design and to Annemette Staal Rasmussen for sharing her expertise on nudging and behavioral design.

# List of abbreviations

HCP, Healthcare professional

AUDIT, Alcohol Use Disorder Identification Test

GP, General practitioner

OPEN, Odense Patient data Explorative Network

# Declarations

## Ethics approval and consent to participate

The Danish College of General Practitioners (MPU 18-2021) and the Research Ethics Committee at the University of Southern Denmark (Case no. 21/66119) approved this study. This study was not eligible for application to the Danish National Research Ethics Committee as it did not include sampling of biological material or collection of sensitive health data, cf. Section 14-2 of the Danish Act on Research Ethics Review of Health Research Projects. The authors state that all experiments and methods in the present study were conducted in accordance with relevant guidelines and regulations.

All participants received oral and written information on the study and signed a written informed consent form on participation prior to study commencement.

## Consent for publication

Not applicable.

## Data availability

Not applicable.

## Disclosure Statement

The authors declare no competing interests.

## Funding

This study was supported by TrygFonden under Grant ID 153248.

The funder had no role in the conduct, analysis, interpretation, reporting, or presentation of the work.

## Authors’ contributions

PNS established the development team and was responsible for participant recruitment and overall workshop facilitation. PNS and the development team planned the workshops and meetings and analyzed the data. PNS drafted the manuscript and prepared tables and figures. ASN, JS, and SR provided feedback throughout the process and oversaw the overall project. ASN, JS, and SR reviewed the manuscript, and all authors approved the final version.

## Supplementary materials

All 15-method material, including example videos, can be accessed at [www.sdu.dk/en/15-metoden](http://www.sdu.dk/en/15-metoden) (material in Danish).

# References

1. World Health Organization. Global status report on alcohol and health 2018: World Health Organization; 2019.

2. Anderson P. Alcohol as a key area. BMJ: British Medical Journal. 1991;303(6805):766.

3. McGovern R, Kaner E. Alcohol prevention and treatment: interventions for hazardous, harmful, and dependent drinkers. The Handbook of Health Behavior Change. 2013:211-27.

4. Rosario F, Santos MI, Angus K, Pas L, Ribeiro C, Fitzgerald N. Factors influencing the implementation of screening and brief interventions for alcohol use in primary care practices: a systematic review using the COM-B system and Theoretical Domains Framework. Implement Sci. 2021;16(1):6.

5. Nilsen P, Aalto M, Bendtsen P, Seppä K. Effectiveness of strategies to implement brief alcohol intervention in primary healthcare. A systematic review. Scand J Prim Health Care. 2006;24(1):5-15.

6. Nilsen P, Andréasson S. Four decades of screening and brief alcohol intervention research: the peg and the hole. Oxford University Press; 2023. p. 3-.

7. Wallhed Finn S. Alcohol dependence: barriers to treatment and new approaches in primary care: Inst för folkhälsovetenskap/Dept of Public Health Sciences; 2018.

8. Johnson M, Jackson R, Guillaume L, Meier P, Goyder E. Barriers and facilitators to implementing screening and brief intervention for alcohol misuse: a systematic review of qualitative evidence. J Public Health (Oxf). 2011;33(3):412-21.

9. Wallhed Finn S, Bakshi A-S, Andréasson S. Alcohol consumption, dependence, and treatment barriers: perceptions among nontreatment seekers with alcohol dependence. Substance Use & Misuse. 2014;49(6):762-9.

10. Wallhed Finn S, Hammarberg A, Andreasson S. Treatment for Alcohol Dependence in Primary Care Compared to Outpatient Specialist Treatment-A Randomized Controlled Trial. Alcohol Alcohol. 2018;53(4):376-85.

11. Wallhed Finn S, Andréasson S, Hammarberg A. Treatment of Alcohol Dependence in Primary Care Compared With Outpatient Specialist Treatment: Twelve-Month Follow-Up of a Randomized Controlled Trial, With Trajectories of Change. J Stud Alcohol Drugs. 2020;81(3):300-10.

12. Skivington K, Matthews L, Simpson SA, Craig P, Baird J, Blazeby JM, et al. A new framework for developing and evaluating complex interventions: update of Medical Research Council guidance. BMJ. 2021;374:n2061.

13. Schøler PN, Søndergaard J, Barfod S, Nielsen AS. Danish feasibility study of a new innovation for treating alcohol disorders in primary care: the 15-method. BMC Primary Care. 2022;23(1):1-14.

14. Schøler PN, Søndergaard J, Rasmussen S, Nielsen AS. Adjusting the 15-method to Danish general practice: identification of barriers, facilitators, and user needs. BMC Prim Care. 2024;25(1):248.

15. Saunders JB, Aasland OG, Babor TF, De La Fuente JR, Grant M. Development of the Alcohol Use Disorders Identification Test (AUDIT): WHO Collaborative Project on Early Detection of Persons with Harmful Alcohol Consumption-II. Addiction (Abingdon, England). 1993;88(6):791-804.

16. Sobell MB, Sobell LC. Stepped care as a heuristic approach to the treatment of alcohol problems. Journal of consulting and clinical psychology. 2000;68(4):573.

17. Miller WR, Rollnick S. Motivational interviewing: Helping people change and grow: Guilford Publications; 2023.

18. Sobell LC, Sobell MB. Timeline follow-back. Measuring alcohol consumption: Springer; 1992. p. 41-72.

19. Raistrick D, Dunbar G, Davidson R. Development of a questionnaire to measure alcohol dependence. British journal of addiction. 1983;78(1):89-95.

20. World Health Organization. The ICD-10 classification of mental and behavioural disorders: clinical descriptions and diagnostic guidelines: World Health Organization; 1992.

21. Sobell MB, Sobell LC. Problem drinkers: Guided self-change treatment: The Guilford Press; 1996.

22. Simonsen J, Robertson T. Routledge international handbook of participatory design: Routledge New York; 2013.

23. Plattner H, Meinel C, Laifer L. Design thinking research: studying co-creation in practice. 1. Aufl.;1;2012; ed. Plattner H, Meinel C, Leifer L, editors. Heidelberg: Springer Verlag; 2012.

24. Bratteteig T. Making change. Dealing with relations between design and use Dr Philos dissertation, Department of Informatics, University of Oslo. 2004.

25. Ehn P, Sjögren D. From system descriptions to scripts for action. Design at work: CRC Press; 2020. p. 241-68.

26. Braten S. Model Monopoly and Communication: Systems Theoretical Notes On Democratization. Acta Sociologica. 1973;16(2):98-107.

27. Borum F, Enderud H. Konflikter i organisationer: belyst ved studier af edb-systemarbejde [Conflicts in organisations, illustrated by cases of computer systems design]. Copenhagen, Denmark: Nyt Nordisk Forlag Arnold Busck. 1981.

28. General practices in the Danish regions [Internet]. Danish Ministry of the Interior and Health and Danish Regions. 2023 [cited 03/10/2023]. Available from: <https://medcom.dk/medcom-in-english/>.

29. Dixon BE, Schmidt T, Nøhr C. Facilitating HIE in Denmark: the story of MedCom, a Danish health information organization. Health Information Exchange: Elsevier; 2023. p. 579-601.

30. Statistics Denmark [Internet]. Statistics Denmark. 2024 [cited 10/10/2024]. Available from: <https://www.dst.dk/en/Statistik/>.

31. General practice and affiliated patients [Internet]. The Danish Health Data Authority. 2023 [cited 10/03/2023]. Available from: <https://sundhedsdatastyrelsen.dk/da/english/health_data_and_registers>

32. Pedersen KM, Andersen JS, Søndergaard J. General practice and primary health care in Denmark. The Journal of the American Board of Family Medicine. 2012;25(Suppl 1):S34-S8.

33. Palinkas LA, Horwitz SM, Green CA, Wisdom JP, Duan N, Hoagwood K. Purposeful Sampling for Qualitative Data Collection and Analysis in Mixed Method Implementation Research. Adm Policy Ment Health. 2015;42(5):533-44.

34. Alkohol og Samfund. Danish interest organization for better alcohol habits 2025 [Available from: <https://alkohologsamfund.dk/>.

35. Region of Southern Denmark. Odense Patient data Explorative Network (OPEN) [Webpage]. 2023 [Available from: <https://en.ouh.dk/research/open-odense-patient-data-explorative-network/>.

36. Schon DA, Wiggins G. Kinds of seeing and their functions in designing. Design Studies. 1992;13(2):135-56.

37. Carroll JM, Kellogg WA, Rosson MB. The task-artifact cycle. Designing interaction: Psychology at the human-computer interface1991. p. 74-102.

38. Leask CF, Sandlund M, Skelton DA, Altenburg TM, Cardon G, Chinapaw MJM, et al. Framework, principles and recommendations for utilising participatory methodologies in the co-creation and evaluation of public health interventions. Res Involv Engagem. 2019;5:2.

39. Clemensen J, Rothmann MJ, Smith AC, Caffery LJ, Danbjorg DB. Participatory design methods in telemedicine research. J Telemed Telecare. 2017;23(9):780-5.

40. Brandt E. Designing exploratory design games: a framework for participation in Participatory Design? Proceedings of the ninth conference on Participatory design: Expanding boundaries in design - Volume 1; Trento, Italy: Association for Computing Machinery; 2006. p. 57–66.

41. Carroll JM, editor Making use: scenarios and scenario-based design. Proceedings of the 3rd conference on Designing interactive systems: processes, practices, methods, and techniques; 2000.

42. Hertzum M, Bansler JP, Havn EC, Simonsen J. Pilot implementation: Learning from field tests in IS development. Communications of the Association for Information Systems. 2012;30(1):20.

43. Davidson R, Raistrick D. The validity of the short alcohol dependence data (SADD) questionnaire: A short self‐report questionnaire for the assessment of alcohol dependence. British Journal of Addiction. 1986;81(2):217-22.

44. Schøler PN, Sondergaard J, Rasmussen S, Volke KH, Nilsen P, Nielsen AS. Determinants of implementing the 15-method in Danish general practice using the consolidated framework for implementation science. Addict Sci Clin Pract. 2025;20(1):43.

45. Anderson P, Kaner E, Wutzke S, Funk M, Heather N, Wensing M, et al. Attitudes and managing alcohol problems in general practice: an interaction analysis based on findings from a WHO collaborative study. Alcohol and Alcoholism. 2004;39(4):351-6.

46. Anderson P, Bendtsen P, Spak F, Reynolds J, Drummond C, Segura L, et al. Improving the delivery of brief interventions for heavy drinking in primary health care: outcome results of the Optimizing Delivery of Health Care Intervention (ODHIN) five-country cluster randomized factorial trial. Addiction. 2016;111(11):1935-45.

47. Katie W, A. HK, R. KH, F. MK, S. HD, E. FD, et al. Clinical Validation of Reduced Alcohol Consumption After Treatment for Alcohol Dependence Using the World Health Organization Risk Drinking Levels. Alcoholism: Clinical and Experimental Research. 2017;41(1):179-86.

48. Witkiewitz K, Kranzler HR, Hallgren KA, O'Malley SS, Falk DE, Litten RZ, et al. Drinking risk level reductions associated with improvements in physical health and quality of life among individuals with alcohol use disorder. Alcoholism: Clinical and Experimental Research. 2018;42(12):2453-65.

49. Witkiewitz K, Falk DE, Litten RZ, Hasin DS, Kranzler HR, Mann KF, et al. Maintenance of World Health Organization Risk Drinking Level Reductions and Posttreatment Functioning Following a Large Alcohol Use Disorder Clinical Trial. Alcoholism: Clinical and Experimental Research. 2019;43(5):979-87.

50. Saunders SM, Zygowicz KM, D'Angelo BR. Person-related and treatment-related barriers to alcohol treatment. J Subst Abuse Treat. 2006;30(3):261-70.

51. S. Mueller MH. Alcohol and Alcohol-related diseases S. Mueller MH, editor: Springer Nature Switzerland AG; 2023.

52. Månsson V, Hårdstedt M, Hammarberg A, Hake A, LoMartire R. Identifying hazardous alcohol use in primary care using phosphatidylethanol: Timing of screening matters. Addiction. 2025.

53. Steensland A, Segernas A, Larsson M, Johansson Capusan A, Kastbom L. B-phosphatidylethanol testing to identify hazardous alcohol use in primary health care-a game changer and a challenge for general practitioners: a qualitative study. Scand J Prim Health Care. 2025;43(2):463-75.

54. Schøler PN, Volke KH, Andréasson S, Rasmussen S, Søndergaard J, Nielsen AS. The identification and treatment of alcohol problems in primary care (iTAPP) study: protocol for a stepped wedge cluster randomized control trial testing the 15-method in a primary care setting. Addiction Science & Clinical Practice. 2024;19(1).

55. Graham Moore MC, Lauren Copeland, Peter Craig, Ani Movsisyan, Pat Hoddinott, Hannah Littlecott, Alicia O’Cathain, Lisa Pfadenhauer, Eva Rehfuess, Jeremy Segrott, Penelope Hawe, Frank Kee, Danielle Couturiaux, Britt Hallingberg, Rhiannon Evans. Adaptation of interventions for implementation and/or re-evaluation in new contexts: The ADAPT guidance (v1.0). 2020.

56. Kirk MA, Moore JE, Wiltsey Stirman S, Birken SA. Towards a comprehensive model for understanding adaptations’ impact: the model for adaptation design and impact (MADI). Implementation Science. 2020;15:1-15.

57. Damschroder LJ, Hagedorn HJ. A guiding framework and approach for implementation research in substance use disorders treatment. Psychology of addictive behaviors. 2011;25(2):194.

58. May CR, Johnson M, Finch T. Implementation, context and complexity. Implement Sci. 2016;11(1):141.

59. Moore G, Campbell M, Copeland L, Craig P, Movsisyan A, Hoddinott P, et al. Adapting interventions to new contexts-the ADAPT guidance. BMJ. 2021;374:n1679.

60. Clemensen J, Larsen SB, Kyng M, Kirkevold M. Participatory design in health sciences: using cooperative experimental methods in developing health services and computer technology. Qualitative health research. 2007;17(1):122-30.

61. Snape D, Kirkham J, Britten N, Froggatt K, Gradinger F, Lobban F, et al. Exploring perceived barriers, drivers, impacts and the need for evaluation of public involvement in health and social care research: a modified Delphi study. BMJ Open. 2014;4(6):e004943.

62. Richards DP, Poirier S, Mohabir V, Proulx L, Robins S, Smith J. Reflections on patient engagement by patient partners: how it can go wrong. Res Involv Engagem. 2023;9(1):41.

63. Sanders EBN, Stappers PJ. Probes, toolkits and prototypes: three approaches to making in codesigning. CoDesign. 2014;10(1):5-14.

64. Star SL, Strauss A. Layers of silence, arenas of voice: The ecology of visible and invisible work. Computer supported cooperative work (CSCW). 1999;8:9-30.

# Tables and figures

**Table 1**. Participant characteristics and attendance in study activities.

| **Participant characteristics** | **Attendance** | | | |
| --- | --- | --- | --- | --- |
|  | **Danish feasibility study of the 15-method** | **Interview*** | **Workshops** | **Field test** |
| GP, male, practice no 1 | ✓ | ✓ | ✓ | ✓ |
| Nurse, female, practice 1 | ✓ | ✓ | ✓ | ✓ |
| GP, female, practice no 2 | ✓ | ✓ | ✓ | ✓ |
| GP, female, practice no 2 | ✓ | ✓ | ✓ | ✓ |
| Nurse, female, practice no 3 |  | ✓ | ✓ |  |
| Nurse, female, practice no 3 |  | ✓ | ✓ |  |
| GP, male, practice no 3 |  | ✓ | ✓ |  |
| GP, female, practice no 3 |  | ✓ | ✓ |  |
| Patient 1, male, no prior or current alcohol-related problems |  | ✓ | ✓ |  |
| Patient 2, female, current hazardous alcohol consumption |  | ✓ |  |  |
| Patient 3, male, no prior or current alcohol-related problems |  | ✓ | ✓ |  |
| Patient 4, male, prior moderate to severe alcohol dependency. |  | ✓ | ✓ |  |
| Patient 5, male, prior severe alcohol dependency |  | ✓ | ✓ |  |
| Project manager, male |  |  | ✓ |  |
| Researcher, male | ✓ | ✓ | ✓ | ✓ |
| Graphic designer, male |  |  | ✓ | ✓ |
| Behavioral psychologist, female |  |  | ✓ | ✓ |

Notes: GP, general practitioner. * conducted prior to the present study and reported elsewhere. Alcohol consumption and alcohol-related problems are self-reported, and patients were free to choose whether they wished to make a statement on their alcohol habits.

**Figure 1**. Study procedure in iterative cycles using the participatory design method Plan, Act, Observe, Reflect (based on Clemensen et al. 2017.)

Notes: WS, Workshop; HCP, Healthcare professional.

**Figure 2**. Picture from workshop one illustrating three parallel sessions in the "dollhouse" at the Plug and Play lab, Health Innovation Centre of Southern Denmark

Notes: Top left: body storming exercise on how to address alcohol habits in a non-judgmental way in a primary care setting. Top right: concept development speed round to envision the optimal future solution for treating alcohol problems in a primary care setting. Bottom left: set-up for a brain-writing exercise on how to best make health care professionals and patients prioritize the time and resources needed to use the 15-method in a general practice setting. Bottom right: graphic design stand-up session focusing on the design and purpose of the 15-method’s material using a Who-What-Where-How matrix.

**Figure 3**. The 15-method quick guide for Danish general practice (translated to English).

Notes: Color coded overview of the *15-*method’s three steps on front page. Infographic on second page (counterphase). Page numbers refer to the patient logbook. “Alkolinjen” is a Danish telephone hotline for anonymous and free counseling on alcohol related problems. The hotline is administered by “Alkohol og Samfund” (Alcohol and Society) an interest organization working for healthier alcohol use in Denmark. Design and graphics by Henrik Nielsen.
